# Political Distinction of Medical Men

**Published:** 1848-08

**Authors:** 


*Political Distinction of Medical Men.*—About thirty members of the National Assembly of France are physicians ; of these M. Buchez, a distinguished writer, was the first President, M. Recurt Vice President, and M. Trélat, Minister of Public Works

In Prussia, ten physicians have been elected to the National Assembly recently organized ; and in Italy, the Pope has appointed the celebrated Italian physician Farini, Under Secretary of State for the papal territories.

